# Mapping the neck disability index to SF-6D in patients with chronic neck pain

**DOI:** 10.1186/s12955-016-0422-x

**Published:** 2016-02-16

**Authors:** Yongjun Zheng, Kun Tang, Le Ye, Zisheng Ai, Bin Wu

**Affiliations:** Department of Pain Management, Huadong Hospital, Fudan University, Shanghai, China; Department of Anesthesiology, Tongren Hospital, School of Medicine, Shanghai Jiaotong University, Shanghai, 200336 China; Department of Pain Management, Renji Hospital, School of Medicine, Shanghai Jiaotong University, Shanghai, 200127 China; Department of Preventive Medicine, College of Medicine, Tongji University, Shanghai, 200092 China; Clinical Outcomes and Economics Group, Department of pharmacy, Ren Ji Hospital, School of Medicine, Shanghai Jiaotong University, Shanghai, 200127 China

**Keywords:** Neck disability index, SF-6D, Model mapping, Chronic neck pain, Utility values

## Abstract

**Background:**

This study sought to statistically map the neck disability index (NDI) to the six-dimension health state short form (SF-6D) to estimate algorithms for use in economic analyses in patients with chronic neck pain (CNP).

**Methods:**

The relationships between NDI and SF-6D scores were estimated by using data from a cohort of patients with chronic neck pain (*n* = 272). By using ordinary least squares (OLS), generalized linear modeling (GLM), censored least absolute deviations (CLAD) and Tobit regression, scores from all 10 items of the NDI instruments were univariately tested against SF-6D values and retained in a multivariate regression model, if statistically significant. The predictive ability of the model was assessed by mean absolute error (MAE), root mean square error (RMSE) and normalized RMSE.

**Results:**

The mean age of the 272 CNP patients was 39.9 ± 12.3 years; 57.8 % of the CNP patients were female. An OLS regression equation that included recreation item of NDI was optimal, with a MAE of 0.04and 0.04 and an RMSE of 0.06and 0.05in the derivation set and validation set, respectively. Predicted utilities accurately represented the observed ones.

**Conclusions:**

We have provided algorithms for the estimation of health state utility values from the response of NDI. Future economic evaluations of the interventions for chronic neck pain could be informed by these algorithms.

## Background

Cost-utility analysis is an increasingly important aspect of health technology assessment, where cost per quality-adjusted life year (QALY) gained is used as a primary endpoint for a novel intervention [1]. The QALY is an estimate of health that represents both survival and health-related quality of life (HRQoL) as a single number [2], where the HRQoL is measured by using a health state utility value, which could be captured by questionnaires. A commonly used preference-based instrument, such as the EuroQol five-dimension (EQ-5D) and the six-dimension health state short form (derived from the short form 36 health survey [SF-36] and the short form 12 health survey [SF-12]) (SF-6D), is used to generate the health-state utility scores [3, 4]. By using health-state utility values, health service researchers can estimate and compare QALYs across different interventions, which can provide decision-making information for healthcare resource allocation [5]. The absence of utility values derived from generic preference-based instruments, however, is a barrier to populating economic models with the best evidence of effectiveness. Statistical regression-based mapping is one method that is gaining popularity when no preference-based instruments are available in the study [6].

The measurement of HRQoL in the pain setting is usually carried out using pain-specific questionnaires rather than generic preference-based instruments because the questionnaires pay attention to relevant health problems and tend to gain more clinically meaningful changes [7, 8]. The most commonly used measurement for evaluating disability in patients with neck pain is the neck disability index (NDI), which comprises a 10-item self-administered questionnaire that was modeled on the Oswestry Back Disability Index by Vernon and Mior in 1991 [9]. Preference-based measures of health status, however, are not always performed.

Neck pain is a common problem with an annual incidence rate ranging from 0.055 cases per 1000 persons (disc herniation with radiculopathy) to 213 cases per 1000 persons (self-reported neck pain) [10, 11]. Nearly two-thirds of adults will experience neck pain during their lives Neck pain is more prevalent among women and middle-aged adults. Some occupations, such as office work, are also risk factors [12]. Due to the necessity of health economic analysis in the area of neck pain, deriving utility values from pain-specific questionnaires is necessary.

Recent studies have shown that there is a strong relationship between the SF-6D and the total NDI scores [13, 14]. However, analysis of mapping items data of NDI to SF-6D in patients with chronic neck pain (CNP) is limited. The aim of the present study was to establish a mapping relationship that uses HRQoL data from the NDI to estimate SF-6D utility values in patients with CNP in order to facilitate economic evaluation of novel therapies for CNP and imply future intervention study of improving utilities by targeting some items.

## Methods

### Data source

Patients with chronic non-specific neck pain were enrolled from the Pain Management Department of Huadong Hospital. The study was done in accordance with the ethical standards of the Declaration of Helsinki, the International Conference on Harmonisation’s good clinical practice, local laws, and applicable regulatory requirements. The study was approved by the Ethics Committee of Huadong Hospital in Shanghai, China. Written informed consent was obtained from all patients before enrollment.

During the first treatment visit after study enrollment, data was collected on demographics and clinical features, as well as across a range of patient-reported outcomes from patient interviews and from two self-administered HRQoL questionnaires: NDI and SF-6D. They also answered questions on socio-demographic information (sex, age, education, housing type and income), self-rated health status (“Compared with past 12 months, what do you think about your present health condition? Better, The same, Worse”) and life style information (e.g. smoking). Eligible patients were aged ≥ 18 years, had neck pain during at least the 3 months prior to the study and had no exercise-related risks. To reduce the potential factor that might interact with neck pain [15], patients were ineligible if they had one or more of the following conditions: existing vestibular pathology; receiving medical intervention in the last 3 months; cervical fracture or dislocation; systemic diseases; neurological, cardiovascular or respiratory disorders affecting physical performance; history of traumatic head injury; inability to provide informed consent; pregnancy.

### Measures

1. Neck-specific disability
  A validated Chinese version of the original 10-item Neck Disability Index (NDI) was employed in this study [16]. The NDI, which is derived from the Oswestry Index and designed for assessing neck pain and disability [17], contains 10 self-reported items: pain intensity, personal care, lifting, reading, headache, concentration, work, driving, sleeping, and recreation [18]. Each item is scored on a 6-point scale from 0 (no disability) to 5 (full disability); the sum score out of all 10 items is calculated using a percentage of the maximal score, with higher values representing greater disability.
2. SF-36/SF-6D
  The SF-36 is a widely used instrument for measuring general health status, and comprises36 self-report questions regarding functional health and well-being [19]. SF-36v2™ was used in this study, and the SF-6D was derived from11 items identified from the SF-36, which comprises six multi-level dimensions of health (physical health problems, bodily pain, general health, vitality, social functioning, role limitations due to emotional problems, and mental health), with each dimension having four to six levels [20]. This classification of health status yields 18,000 possible health state scores, which can range from 0 to 1.0, where 0 indicates the worst health state and 1.0 the best health state. The utility score can be used in the health economic evaluation of interventions and for population health surveys [21, 22]. The Hong Kong Chinese version and HK scoring algorithm of SF-6D was not adopted because it was not widely validated as United Kingdom version and also used less health states in developing the algorithm [23].

### Statistical methods

The SF-6D index scores were regressed onto the individual item scores of NDI by using the ordinary least squares (OLS) regression with the suggestion that more complex models may not add predictive power or reduce errors in prediction [24]. The scores for individual and all items in the NDI in turn were offered to the model to give a general algorithm for predicting SF-6D scores:

1 $$ \mathrm{S}\mathrm{F}\hbox{-} 6\mathrm{D}\kern0.5em \mathrm{score}=\alpha +\Sigma \beta \times \left( Scale\kern0.5em  score\right) $$

All variables were treated as continuous variables. Backward stepwise selection with a significance level of 0.1 from the full model was used to identify statistically significant variables.

When data have pronounced ceiling effects, which is a common phenomenon observed in both health profile and preference-based measures, the use of OLS regression violates the statistical requirement for linearity of conditional expectation, leading to inaccurate predictions of preference-based scores and inaccurate identification of predictor variables. To address this ceiling effects and reduce the potential estimation errors of OLS method, generalized linear modeling (GLM) [25], Censored Least Absolute Deviations (CLAD) and the Tobit model were used to provide an extensional analysis [26].

Because the main purpose of a mapping study is to derive an algorithm that accurately derives health state utility values from other data sets, mean absolute error (MAE) and root-mean-squared error (RMSE) were employed to assess the goodness of fit of the models according to external guidance [6]. RMSE was normalized to the range of score forSF-6D and expressed as % RMSE [27]. The MAE is the mean of absolute differences between the observed SF-6D utility score and the SF-6D utility score predicted from the model; the RMSE is the positive square root of the mean squared estimation error between the observed and predicted values. Smaller MAE and RMSE values suggest better model performance. Fit was decided based on lowest RMSE. The best-fitting model(s) were then re-estimated using data for the validation cohort. External validation would be conducted based on the following equations between total NDI score and SF-6D utilities [13, 14].

2 $$ \mathrm{S}\mathrm{F}\hbox{-} 6\mathrm{D}\kern0.5em \mathrm{score}=\hbox{-} 0.0115* Total\kern0.5em  scores\kern0.5em  of\kern0.5em NDI+0.8383 $$

3 $$ \mathrm{S}\mathrm{F}\hbox{-} 6\mathrm{D}\kern0.5em \mathrm{score}=\hbox{-} 0.0135* Total\kern0.5em  scores\kern0.5em  of\kern0.5em NDI+0.8686 $$

4 $$ \mathrm{S}\mathrm{F}\hbox{-} 6\mathrm{D}\kern0.5em \mathrm{score}=\hbox{-} 0.0115* Total\kern0.5em  scores\kern0.5em  of\kern0.5em NDI+0.8391 $$

5 $$ \mathrm{S}\mathrm{F}\hbox{-} 6\mathrm{D}\kern0.5em \mathrm{score}=\hbox{-} 0.01005* Total\kern0.5em  scores\kern0.5em  of\kern0.5em NDI+0.78219 $$

Due the absence of external data sets, external validation samples were created by randomly selecting half the derivation and validation sample [27]. Statistical analyses were carried out using the statistical programming environment R (R Development Core Team, 2014). *P* < 0.05 was considered statistically significant.

## Results

### Patient characteristics, HRQoL scores, and utility values

The study enrolled 272 patients (180 randomly assigned into the derivation set and 92 in the validation set). Baseline patient characteristics are listed in Table 1. Just over one half (57.8 %) of the respondents in the whole set were female; the average (± standard deviation [SD]) age at enrollment was 39.9 ± 12.3 years; time from diagnosis was 27.4 ± 7.37 months; nearly 45 % were college-educated. There were no significant differences in the derivation and validation sets across all variables.

**Table 1:** Descriptive statistics for the whole, derivation and validation sets

Characteristic	Whole set (*n* = 272)	Derivation set (*n* = 180)	Validation set (*n* = 92)
Age (year): mean (SD)	39.9 (12.3)	39.8 (12.4)	40.3 (11.4)
Female (%)	57.8 %	60.0 %	52.9 %
BMI(kg/m^2^): mean (SD)	21.3 (3.17)	21.4 (3.2)	21 (3.1)
College education (%)	44.0 %	45.8 %	42.9 %
Disease duration (months): mean (SD)	27.4 (7.37)	27.7 (7.45)	27 (7.2)
Physical activity ≥3 times a week	21.1 %	21.0 %	21.3 %
Smoking (%)	17.2 %	17.7 %	16.2 %
Working with computer >6 hours a day (%)	44.2 %	44.5 %	43.5 %

Table 2 shows the summary characteristics of the outcome measures across the whole, estimation and validation sets. In the whole set, the average utility value of SF-6D was 0.519 ± 0.092 and the inter quartile range (IQR) was 0.454 to 0.563. The minimum observed utility value was 0.19 and the maximum was 0.82. The mean NDI Global score was 28.46 ± 12.93 and the IQR was 18 to 40. The mean pain intensity score was near 3.5, which was higher than other item scores. Personal care item had the lowest average scores (2.14). The total scores of the ten NDI items were similar between derivation and validation sets.

**Table 2:** Summary characteristics of outcome measures across the samples

Characteristic	Whole set (*n* = 272)		Derivation set (*n* = 180)		Validation set (*n* = 92)	
	Mean (SD)	Median (IQR)	Mean (SD)	Median (IQR)	Mean (SD)	Median (IQR)
SF-6D	0.52 (0.09)	0.49 (0.45-0.56)	0.53 (0.09)	0.49 (0.47-0.58)	0.5 (0.09)	0.48 (0.45-0.55)
NDI global scores	28.46 (12.93)	27 (18-40)	28.98 (13.87)	27.5 (18-43)	27.4 (10.97)	27 (18-34.75)
Scores of 10 individual items						
Pain intensity	3.47 (1.55)	3 (2-5)	3.52 (1.62)	3 (2-5)	3.37 (1.43)	3 (2-5)
Personal care	2.14 (1.02)	2 (2-3)	2.17 (1.04)	2 (2-3)	2.1 (1)	2 (1.25-3)
Lifting	2.63 (1.47)	2 (2-4)	2.7 (1.45)	2 (2-4)	2.5(1.5)	2 (1-3.75)
Reading	3.08 (1.38)	3 (2-4)	3.12 (1.45)	3 (2-4)	3 (1.26)	3 (2-4)
Headaches	2.67 (1.97)	2 (1-4)	2.68 (2.02)	2 (1-5)	2.63 (1.9)	2 (1-3.75)
Concentration	2.94 (1.89)	2 (1-5)	3.02 (1.9)	2.5 (1-5)	2.8 (1.88)	2 (1-4)
Work	2.74 (1.73)	2.5 (2-3)	2.77 (1.86)	2 (2-5)	2.7 (1.44)	3 (1.25-3)
Driving	2.97 (1.88)	3 (2-5)	3.08 (1.94)	3 (2-5)	2.73 (1.74)	2.5 (2-4)
Sleeping	2.58 (1.62)	2 (1-4)	2.72 (1.65)	2 (1-4)	2.3 (1.56)	2 (1-3.75)
Recreation	3.23 (1.64)	3 (2-5)	3.22 (1.76)	3 (2-5)	3.27 (1.41)	3.5 (2-4)

### Mapping NDI to SF-6D

Results of the OLS regression analysis are summarized in Table 3. In the trimmed model (i.e., the backward elimination model), recreation item showed statistical significance. The pain intensity and sleeping item, which had a p-value of 0.0146 and 0.0064 in the full model, became statistically insignificant in the trimmed model, in which it had a p-value of 0.2 and 0.45, respectively. Recreation Recreation was the most influential item in both models (Table 3). Results of the GLM, Tobit and CLAD method regression analysis are presented in Appendix Tables 5-7.

**Table 3:** Simple correlations and multiple regression multiple regression analyses mapping NDI data to EQ-5D

Predictors	Full model		Trimmed model	
	Estimate (β)	*p*-value	Estimate (β)	*p*-value
Total score of 10 items				
Intercept	0.5927	<0.0001	NA	NA
Total score	-0.0026	0.0004	NA	NA
Pain intensity item				
Intercept	0.5587	<0.0001	NA	NA
Score	-0.0115	0.0651	NA	NA
Personal care item				
Intercept	0.5963	<0.0001	NA	NA
Score	-0.0362	0.0087	NA	NA
Lifting item				
Intercept	0.5591	<0.0001	NA	NA
Score	-0.0154	0.0198	NA	NA
Reading item				
Intercept	0.5727	<0.0001	NA	NA
Score	-0.0176	0.0116	NA	NA
Headaches item				
Intercept	0.5488	<0.0001	NA	NA
Score	-0.0113	0.021	NA	NA
Concentration item				
Intercept	0.5663	<0.0001	NA	NA
Score	-0.0162	0.0013	NA	NA
Work item				
Intercept	0.5797	<0.0001	NA	NA
Score	-0.0222	<0.0001	NA	NA
Driving item				
Intercept	0.565	<0.0001	NA	NA
Score	-0.0156	0.0022	NA	NA
Sleeping item				
Intercept	0.5342	<0.0001	NA	NA
Score	-0.006	0.3195	NA	NA
Recreation item				
Intercept	0.5957	<0.0001	NA	NA
Score	-0.0238	<0.0001	NA	NA
All items in one model				
Intercept	0.5827	<0.0001	0.5957	<0.0001
Pain intensity	0.0256	0.0146	-0.0315	0.2414
Personal care (Washing, Dressing, etc.)^a^	-0.021	0.1007	NS	NS
Lifting	0.0014	0.8741	NS	NS
Reading	-0.0009	0.9314	NS	NS
Headaches	-0.0013	0.8623	NS	NS
Concentration	-0.0102	0.3431	NS	NS
Work	-0.0113	0.3347	NS	NS
Driving	0.0049	0.5255	NS	NS
Sleeping^a^	0.021	0.0064	0.023	0.4514
Recreation	-0.0348	0.0008	-0.0238	<0.0001

*NS* not significant, *NA* not applicable

^a^The item would be removed in the final trimmed model due to no significance

Table 4 shows the model performance for both the derivation and validation sets when the trimmed model was fitted. The MAE values for the derivation and validation sets were 0.0404 ± 0.033and 0.0404 ± 0.030030, respectively. The normalized RMSE were 0.06 and 0.0505, respectively. The MAEs and RMSEs estimated by the GLM, Tobit and CLAD method was 0.06 and 0.07, 0.06 and 0.07 and 0.05 and 0.06, respectively, which offered no advantage in terms of estimation errors over the OLS models.

**Table 4:** Performance in trimmed model according to EQ-5D quartile in the derivation and validation sets

	Derivation set (*n* = 180)		Validation set (*n* = 92)	
	Observed values	Predictive values	Observed values	Predictive values
Mean(SD)	0.53 (0.09)	0.52 (0.04)	0.5 (0.09)	0.52 (0.04)
Min	0.21	0.29	0.19	0.33
P25	0.45	0.46	0.46	0.49
Median	0.48	0.52	0.50	0.52
P75	0.56	0.57	0.59	0.56
Max	0.82	0.68	0.73	0.63
Adjusted R^2^	0.49		0.56	
MAE (SD)	0.04 (0.033)		0.04 (0.030)	
RMSE (normalized for range)	0.06 (8.6 %)		0.05 (7.2 %)	

**Fig. 1:**
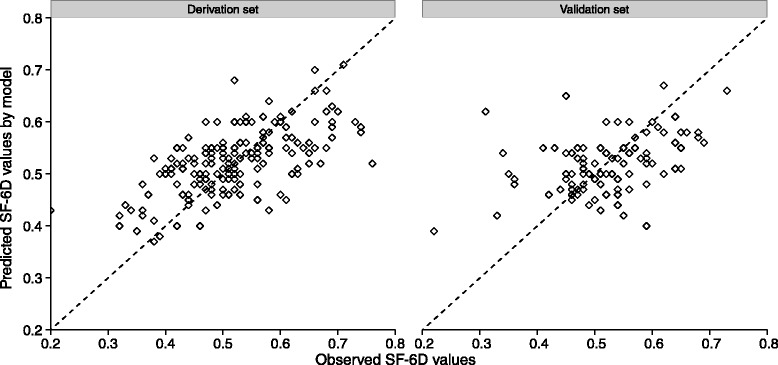
Scatter plot of predicted values based on the trimmed model parameters versus the observed SF-6D values. A perfect fit is indicated by the 45° reference dotted-line. The blue line is the shown as the liner fit line

We not only examined accuracy of the model but also the distributions of the predicted scores. The observed mean value of the SF-6D score was similar to the predicted SF-6Dindexes of both sets (Table 4). Both sets reported lower variability across predicted utility values with similar SDs at 67 % of the magnitude of the observed SF-6D scores. In both data sets, the 25th percentile and median predicted values were overestimated, but 75th percentile predicted values were underestimated. A plot of observed SF-6D index versus predicted utility scores from the trimmed model indicates that the model fits the data well in both the derivation and validation sets (Fig. 1); the Pearson correlation coefficients were 0.70and 0.7575, respectively.

### External validation

The MAEs estimated by equation 2, 3, 4 and 5 were 0.11, 0.13, 0.11 and 0.10, and RMSE were 0.14, 0.17, 0.14 and 0.13, respectively. When the algorithm mapped our total scores to SF-6D utilities was used (Table 3), the MAE and RMSE were 0.07 and 0.08, respectively.

## Discussion

This study explored an algorithm for linking the NDI to the SF-6D using regression equations to predict SF-6D utility values in a data set of patients with CNP. We found that in settings where the NDI, but not SF-6D, is collected, estimating the SF-6D from the NDI score using an algorithm appears useful. The trimmed model with recreation item as explanatory variables has relatively lower MAE and RMSE in comparison with the full model. We noticed that the mean age of our CNP patients were not old (Table 1), recreation might have a considerable impact on their health quality of life because it has an indispensable role in their life [28]. Other mapping studies also found that recreation could notably affect the utility value [29, 30]. This finding indicates that any treatment of improving the recreation might be appreciated in patients with CNP. To the best of our knowledge, the current analysis is the first study to develop the mapping algorithm from NDI items to the SF-6D in patients with CNP; this gives our predictive model the advantage of being applicable to Chinese patients with CNP. Because of the significant loss of data when derived method was used, health preference should be measured directly. However, mapping from condition-specific measure was also reasonable because the mapped score has been pone to have greater extent of sensitivity and responsiveness in patients with different patient populations [31].

Comparing predictions of the preference-based scores using NDI items suggested that a fairly good performance was found for the SF-6D in both the derivation (MAE = 0.0404, RMSE = 0.0606) and validation sets (MAE = 0.0404, RMSE = 0.0505), although the adjusted R^2^ was 0.49and 0.5656, respectively. The performance of validation sets was relatively better than derivation sets, which might be caused by the stronger convergence of former comparing with latter. Brazier JE and colleagues found that the fit of an algorithm mapping a condition-specific instrument to SF-6D is variable because the values of R^2^ ranged from 0.17 to 0.51 in the other studies. Because the purpose of a mapping algorithm is to predict health state utility values from other datasets, however, the predictive accuracy of the model should be examined [32]. R^2^ and adjusted R^2^ used as measures of explanatory power do not have enough information for testing model performance of a mapping algorithm; they also do not indicate if the mapping algorithm is suitable for the entire dataset [6]. Consequently, the quality of performance of the models used in this mapping analysis is likely to be in line with the typical model performance found in other mapping studies.

Similar to other studies [33–35], our finding indicates that the mapping equations overestimated the utilities for the severe health states, whereas they underestimated those for the mild health states (Fig. 1). The final mapping model resulted in the best predicted performance with utility scores ranging from 0.45 to 0.56 (Table 2). It is unknown why this is the case, but it lies in other factors which were sensitive to a patient’s quality of life that may be captured in health-preference instruments but may not be captured in NDI. Because almost half of the enrolled patients fell into this category when they were first diagnosed with CNP, it was expected that the model would perform well in predicting the utilities of these patients. Nevertheless, because of the low MAE and RMSE values, the final model could be recommended as an acceptable method for estimating utilities from the NDI responses for use in a cost-utility study.

The previous two studies translated NDI total scores into SF-6D indexes by using linear regression modeling [13, 14]. Our cohort had similar overall mean SF-6D indexes and NDI total scores in comparison with the sample reported by Richardson SS and colleagues (SF-6D at 0.49 and NDI total scores at 28.54), which was notably different from the data published by Carreon LY and colleagues (SF-6D at 0.67 and NDI total scores at 14.55), possibly due to the study setting and population. Their results showed that the correlations between NDI and SF-6D utility scores were strong and statistically significant, which provides some face validity to the relationships with the SF-6D observed in our study. However, external validation found that the MAEs and RMSEs estimated by using their equation were higher than ours. One of the potential reason is the different characteristics of the patient cohorts, including the ethnic and diseases Another important reason might be the different predictors because we found that model using the total score as the predictor had a relatively high MAE and RMSE in comparison with model using the individual items.

No known studies have compared and mapped the individual items of NDI and SF-6D in patients with CNP, but a recent study by Carreon LY and colleagues compared the NDI with the EQ-5D-3 L in patients with neck and/or upper-extremity complaints [36]. They found no significant relationship between the EQ-5D-3 L and the NDI to allow for a valid estimation of EQ-5D-3 L indexes from the NDI using regression modeling, although the model using the individual NDI items had an R^2^ of 0.46 and an RMSE of 0.172. The authors think the reason is that the different descriptive components of the EQ-5D-3 L and the NDI are used for measuring very different constructs. Each dimension of EQ-5D-5 L has 5 levels instead of 3 [37], which might establish a stronger and more robust relationship with the NDI, allowing for prediction of the EQ-5D indexes from the NDI.

There are some weaknesses in this study. First, the final model equation might not generalize as well to the NDI scores of the patients with neck disease other than CNP because the external validation found the performance of the equations derived from the different disease cohort was different. How well the model will generalize to other morbidities related to neck pain should be considered, and validation is necessary. Second, for the purpose of the present analysis, we currently do not have enough information regarding other health outcomes or comorbidities to analyze in the models, such as the severity of the CNP due to the small sample size. Using these data as covariates would improve the performance of the models. Future work can include more comprehensive demographic data from patient records. Third, due to the cross-sectional nature of the dataset used in the present study, it is unclear whether the predictive model reported in this study will change over time. No other independent dataset with observations on both SF-6D and NDI could be used to evaluate the external validity of the mapping algorithms reported by this study. There’s also the point that since the Chinese specific scoring algorithm for SF-6D is not available yet, the original UK scoring algorithm has been employed in the study, which is the most widely used methods. The differences of the background characteristics in the two populations might have some influences on the results. Finally, Over-/under-prediction on the bottom/top SF-6D utilities might limits the wide usage of the algorithm, which might be more suitable in CNP patients with moderate pain. Hence, future analyses are necessary to test the accuracy of our methods by using data obtained elsewhere [4].

## Conclusions

In conclusion, the statistical performance of the final models demonstrated that it is possible to estimate health state utility values for SF-6D from NDI. Due to the limitations in the current analysis, further research might be required to update the model and examine the performance in other ethnic populations.

## Appendix

**Table 5:** Simple correlations and multiple regression multiple regression analyses mapping NDI data to EQ-5D with GLM method

Predictors	Full model		Trimmed model	
	Estimate (β)	*p*-value	Estimate (β)	*p*-value
Total score of 10 items				
Intercept	0.558685	<0.0001	NA	NA
Total score	-0.002599	0.000378	NA	NA
Pain intensity item				
Intercept	0.5587	<0.0001	NA	NA
Score	-0.011534	0.0651	NA	NA
Personal care item				
Intercept	0.5963	<0.0001	NA	NA
Score	-0.03619	<0.0001	NA	NA
Lifting item				
Intercept	0.5591	<0.0001	NA	NA
Score	-0.015351	0.0198	NA	NA
Reading item				
Intercept	0.5727	<0.0001	NA	NA
Score	-0.01755	0.0116	NA	NA
Headaches item				
Intercept	0.5488	<0.0001	NA	NA
Score	-0.011296	0.021	NA	NA
Concentration item				
Intercept	0.5663	<0.0001	NA	NA
Score	-0.016173	0.00135	NA	NA
Work item				
Intercept	0.5797	<0.0001	NA	NA
Score	-0.022237	<0.0001	NA	NA
Driving item				
Intercept	0.565	<0.0001	NA	NA
Score	-0.015599	0.00218	NA	NA
Sleeping item				
Intercept	0.5342	<0.0001	NA	NA
Score	-0.005999	0.32	NA	NA
Recreation item				
Intercept	0.5957	<0.0001	NA	NA
Score	-0.02382	<0.0001	NA	NA
All items in one model				
Intercept	0.5827	<0.0001	0.601765	<0.0001
Pain intensity	0.0256	0.014603164	0.014551	0.04971
Personal care (Washing, Dressing, etc.)	-0.0210	0.100652028	-0.026856	0.00422
Lifting	0.0014	0.874139856	NS	NS
Reading	-0.0009	0.93140086	NS	NS
Headaches	-0.0013	0.862284178	NS	NS
Concentration	-0.0102	0.343063323	NS	NS
Work	-0.0113	0.334658954	NS	NS
Driving	0.0049	0.525538472	NS	NS
Sleeping^a^	0.0210	0.006355019	0.020376	0.00482
Recreation	-0.0348	0.000773979	-0.0238	<0.0001

*NS* not significant, *NA* not applicable

^a^The item would be removed in the final trimmed model due to no significance

**Table 6:** Simple correlations and multiple regression multiple regression analyses mapping NDI data to EQ-5D with CLAD method

Predictors	Full model		Trimmed model	
	Estimate (β)	*p*-value	Estimate (β)	*p*-value
Total score of 10 items				
Intercept	0.5927	<0.0001	NA	NA
Total score	-0.002599	0.0003276	NA	NA
Pain intensity item				
Intercept	0.5587	<0.0001	NA	NA
Score	-0.01153	0.0622	NA	NA
Personal care item				
Intercept	0.5963	<0.0001	NA	NA
Score	-0.03619	<0.0001	NA	NA
Lifting item				
Intercept	0.5591	<0.0001	NA	NA
Score	-0.01535	0.01851	NA	NA
Reading item				
Intercept	0.5727	<0.0001	NA	NA
Score	-0.01755	0.0116	NA	NA
Headaches item				
Intercept	0.5727	<0.0001	NA	NA
Score	-0.01755	0.01074	NA	NA
Concentration item				
Intercept	0.5488	<0.0001	NA	NA
Score	-0.0113	0.01967	NA	NA
Work item				
Intercept	0.5663	<0.0001	NA	NA
Score	-0.01617	0.001198	NA	NA
Driving item				
Intercept	0.58	<0.0001	NA	NA
Score	-0.0222	0.00195	NA	NA
Sleeping item				
Intercept	0.534	<0.0001	NA	NA
Score	-0.006	0.314	NA	NA
Recreation item				
Intercept	0.5957	<0.0001	NA	NA
Score	-0.02382	<0.0001	NA	NA
All items in one model				
Intercept	0.5827	<0.0001	0.60176522	<0.0001
Pain intensity	0.0256	0.009326	0.01455149	0.04359
Personal care (Washing, Dressing, etc.)	-0.0210	0.08008	-0.02685645	0.003282
Lifting	0.0014	0.8657	NS	NS
Reading	-0.0009	0.9268	NS	NS
Headaches	-0.0013	0.8531	NS	NS
Concentration	-0.0102	0.3117	NS	NS
Work	-0.0113	0.3033	NS	NS
Driving	0.0049	0.4981	NS	NS
Sleeping^a^	0.0210	0.003691	0.02037582	0.003771
Recreation	-0.0348	0.0003551	-0.03972382	<0.0001

*NS* not significant, *NA* not applicable

^a^The item would be removed in the final trimmed model due to no significance

**Table 7:** Simple correlations and multiple regression multiple regression analyses mapping NDI data to EQ-5D with Tobit method

Predictors	Full model		Trimmed model	
	Estimate (β)	*p*-value	Estimate (β)	*p*-value
Total score of 10 items				
Intercept	0.5926677	<0.0001	NA	NA
Total score	-0.0025993	0.000184	NA	NA
Pain intensity item				
Intercept	0.558685	<0.0001	NA	NA
Score	-0.011534	0.0589	NA	NA
Personal care item				
Intercept	0.596304	<0.0001	NA	NA
Score	-0.036187	<0.0001	NA	NA
Lifting item				
Intercept	0.559125	<0.0001	NA	NA
Score	-0.015351	0.0164	NA	NA
Reading item				
Intercept	0.572718	<0.0001	NA	NA
Score	-0.01755	0.00914	NA	NA
Headaches item				
Intercept	0.548824	<0.0001	NA	NA
Score	-0.011296	0.0175	NA	NA
Concentration item				
Intercept	0.56632	<0.0001	NA	NA
Score	-0.01617	0.000813	NA	NA
Work item				
Intercept	0.57973	<0.0001	NA	NA
Score	-0.022237	<0.0001	NA	NA
Driving item				
Intercept	0.56498	<0.0001	NA	NA
Score	-0.015599	0.0014	NA	NA
Sleeping item				
Intercept	0.534168	<0.0001	NA	NA
Score	-0.005999	0.311	NA	NA
Recreation item				
Intercept	0.59572	<0.0001	NA	NA
Score	-0.02382	<0.0001	NA	NA
All items in one model				
Intercept	0.5827	<0.0001	0.575353	<0.0001
Pain intensity^a^	0.0256	0.007693919	0.009556	0.18758
Personal care (Washing, Dressing, etc.)	-0.0210	0.076227642	NS	NS
Lifting	0.0014	0.865308708	NS	NS
Reading	-0.0009	0.926560041	NS	NS
Headaches	-0.0013	0.852636711	NS	NS
Concentration	-0.0102	0.30862753	NS	NS
Work	-0.0113	0.30016401	NS	NS
Driving	0.0049	0.496119248	NS	NS
Sleeping	0.0214	0.002766939	0.021422	0.00281
Recreation	-0.0448	0.000189112	-0.03972382	<0.0001

*NS* not significant, *NA* not applicable

^a^The item would be removed in the final trimmed model due to no significance
